# Integrated Metabolomics and Transcriptomics Reveal Metabolic Patterns in Retina of STZ-Induced Diabetic Retinopathy Mouse Model

**DOI:** 10.3390/metabo12121245

**Published:** 2022-12-09

**Authors:** Ruonan Wang, Qizhi Jian, Guangyi Hu, Rui Du, Xun Xu, Fang Zhang

**Affiliations:** 1 Department of Ophthalmology, Shanghai General Hospital, Shanghai Jiao Tong University School of Medicine, Shanghai 200080, China; 2 National Clinical Research Center for Eye Diseases, Shanghai General Hospital, Shanghai Jiao Tong University School of Medicine, Shanghai 200080, China; 3 Shanghai Key Laboratory of Ocular Fundus Diseases, Shanghai 200080, China; 4 Shanghai Engineering Center for Visual Science and Photomedicine, Shanghai 200080, China; 5 Shanghai Engineering Center for Precise Diagnosis and Treatment of Eye Diseases, Shanghai 200080, China; 6 Department of Endocrinology and Metabolism, Shanghai General Hospital, Shanghai Jiao Tong University School of Medicine, Shanghai 200080, China

**Keywords:** diabetic retinopathy, retina, metabolomics, transcriptomics

## Abstract

Diabetic retinopathy (DR), as the leading cause of vision loss in the working-age population, exhibits unique metabolite profiles in human plasma and vitreous. However, those in retina are not fully understood. Here, we utilized liquid and gas chromatography–tandem mass spectrometry technology to explore metabolite characteristics of streptozotocin (STZ)-induced diabetic mice retina. A total of 145 metabolites differed significantly in diabetic retinas compared with controls. These metabolites are mainly enriched in the Warburg effect, and valine, leucine and isoleucine degradation pathways. To further identify underlying regulators, RNA sequencing was performed to integrate metabolic enzyme alterations with metabolomics in STZ-induced diabetic retina. Retinol metabolism and tryptophan metabolism are the shared pathways enriched by metabolome and transcriptome. Additionally, transcriptomic analysis identified 71 differentially expressed enzyme-related genes including Hk2, Slc7a5, Aldh1a3 and Tph integrated with altered metabolic pathways. In addition, single nucleotide polymorphisms within 6 out of 71 genes are associated with increased diabetes risk. This study lays the foundation for mechanism research and the therapeutic target development of DR.

## 1. Introduction

Diabetic retinopathy (DR), the leading cause of preventable blindness in the working-age population worldwide, is one of the most common complications of diabetes mellitus (DM) [1,2]. With the development of the economy and the increase in life expectancy, the global population of diabetes and DR is expected to expand further [3]. However, therapeutic approaches are limited since the pathology is not fully understood.

Metabolic abnormalities of diabetic retinopathy have been reported to be associated with functional and structural alterations in preclinical models and humans [4]. They may provide potential therapeutic targets. Metabolomics, a research method for qualitative and quantitative analysis of small-molecule metabolites, is an emerging technology for the study of potential biomarkers and therapeutic approaches underlying metabolic diseases [5]. Several studies on the metabolomics of blood or vitreous of DR patients have been performed [6]. For example, Xuan et al. used a multiplatform to measure the serum metabolic profile of 905 patients with diabetes without DR and with DR, and revealed novel serum metabolite biomarkers for DR including 12-hydroxyeicosatetraenoic acid (12-HETE) and 2-piperidone [7]. Profound changes in several metabolites were observed in the plasma of DR patients, including cytidine [8], adenosine [9], glutamine [10] and pseudouridine [11]. Moreover, the metabolomics study suggested that creatine levels were decreased in the vitreous humor of proliferative diabetic retinopathy (PDR) patients compared to control participants [12]. In addition, arginine and proline metabolism [12], valine, leucine, and isoleucine biosynthesis [13], and the pentose phosphate pathway [14] were found to be dysregulated in the diabetic retinopathy vitreous humor and were considered to be associated with DR. However, despite great advances in the metabolomics profiling of blood and vitreous in DR patients, there is little access to the routine surgical collection of the retinas of DR patients. Only one study reported that the synthesis of complex lipids was diminished and the mitochondrial β-oxidation of fatty acids was impaired in the postmortem retinas of patients with DR [15].

Mice, whose genome is 90% identical to the human genome, provide considerable promise as models to investigate the pathogenesis of diseases due to the availability of molecular tools. According to previous studies, the phenotype of diabetic retinopathy was observed in STZ mice, including increased numbers of astrocytes and gliosis, retinal inner nuclear layer (INL) and outer nuclear layer (ONL) thinning, and acellular capillaries and pericyte ghosts [16,17]. Thus, mice are a reliable alternative source of retinas, while limited reports of metabolism in the retina of diabetic animal models have been described. Sas et al. examined the lipid levels in plasma and retinas of db/db mice as type 2 diabetes models and observed 61 distinct lipid abnormalities in diabetic retinas [18]. Another study evaluated the pharmacodynamic effects of a traditional Chinese medicine by measuring the untargeted metabolomics of retinas of streptozotocin (STZ)-induced DR rats [19]. However, the metabolic features of diabetic retina are not fully understood.

This study applied untargeted metabolomics by ultra-high performance liquid chromatography–tandem mass spectrometry (UHPLC-MS/MS) and gas chromatography–mass spectrometry (GC-MS) to identify the metabolic characteristics of STZ-induced diabetic retina. We also performed RNA sequencing of diabetic retinas to integrate metabolic enzyme changes at transcriptional levels with the metabolomics analysis, which may contribute to further revealing the pathogenesis of DR and identifying targets for intervention and treatment.

## 2. Materials and Methods

### 2.1. Animals

C57BL/6J male mice at 8–10 weeks old were intraperitoneally injected with streptozotocin (Sigma, Saint Louis, MO, USA, cat. no. V900890) to induce diabetes at 55 mg/kg for 5 consecutive days, as previously described [20]. Blood glucose levels and animal fasting weight were measured every week after STZ administration by electronic balance (Sartorius, Goettingen, NI, Germany) and the Accu-Chek active glucometer (Roche, Basel, BS, Switzerland). Only mice with blood glucose levels higher than 300 mg/dL were included in the diabetic group and used for further study. The mice that sustained high blood glucose levels for 16 weeks were sacrificed to obtain retina tissue for metabolomics analysis and transcriptome sequencing. All animal experiments were performed in accordance with the relevant ethical regulations of Shanghai General Hospital. The study was approved by the Animal Care and Use Committee of Shanghai General Hospital.

### 2.2. Optical Coherence Tomography (OCT)

OCT images of mice at 15 weeks post-last injection were acquired using a Phoenix Research Lab system (Phoenix MICRON, Bend, OR, USA), as previously described [21]. A total of 10 B scans of retina were captured and averaged to produce the final OCT images. InSight Software version 2.0 (Phoenix MICRON, Bend, OR, USA) was used to analyze the scans, and measurements were performed 200 μm, 300 μm and 400 μm away from the optic disc. The thickness of the inner retina layer was measured.

### 2.3. Isolation of Retinas

Eyes from wild type and STZ-induced diabetic mice were enucleated and transferred in a dish with normal saline. Under a dissecting microscope, an excision was carefully performed along the limbus to expose the lens. With the posterior eye cup exposed, the retina was gently peeled off the underlying retinal pigmented epithelium. The retina from each eye was collected in a tube, flash-frozen in liquid nitrogen, and stored at −80 °C until further processing.

### 2.4. Non-Targeted Metabolomics by UHPLC-MS/MS

The retinas of six STZ-induced diabetic mice and nine healthy mice were homogenized in a 40-fold volume of cold methanol/water/Methyl tert-butyl ether (MTBE) (32:8:10, *v*/*v*/*v*) by using a Tissue Lyser (JX-24, Jingxin, Shanghai, China) with zirconia beads for 3 min at 40 Hz. The homogenate was vortexed for 1 min and incubated at −20 °C for 30 min. After centrifugation at 14,000× *g* for 15 min at 4 °C, 100 μL of supernatants was evaporated to dryness. The dry residues were reconstituted in 50 μL of 50% aqueous acetonitrile (1:1, *v*/*v*).

Chromatographic separation was performed on the Ultimate 3000 UHPLC system (Thermo Fisher Scientific, Waltham, MA, USA) with a BEH Amide column (2.1 mm × 100 mm, 1.7 μm; Waters Co., Milford, MA, USA) at 40 °C. For positive mode, the mobile phases consisted of (phase A) water and (phase B) acetonitrile/water (95:5, *v*/*v*), both with 10 mM ammonium acetate and 0.1% formic acid. The linear elution program was applied as the following gradient: 0 min, 90%B; 6 min, 80%B; 11 min, 50%B and held until finally returned to 90%B at 12.5 min and held to 15 min. For negative mode, the mobile phases consisted of (phase A) water and (phase B) acetonitrile/water (90:10, *v*/*v*), both with 15 mM ammonium formate (pH = 9, modified by ammonium hydroxide). The flow rate was 0.35 mL/min with the following linear gradient: 0 min, 95%B; 5 min, 80%B; 10 min, 75%B; 11 min, 55%B and held to 12.5 min; 13 min, 95%B and held to 17 min. The injection volume was 2 μL and 3 μL for positive mode and negative mode, respectively.

The eluents were analyzed on the Q-Exactive MS (Thermo Fisher Scientific, Waltham, MA, USA) in Heated Electrospray Ionization Positive (HESI+) and Negative (HESI−) mode separately. Acquisition settings were as follows: spray voltage, 4000 V for HESI+ and 3500 V for HESI−; capillary temperature, 320 °C and 320 °C for positive mode, 250 °C and 300 °C for negative mode; sheath gas, 35 (Arb, arbitrary unit); aux gas, 10 (Arb); and S-Lens RF Level, 50 (Arb). The full scan was performed at a high-resolution of 70,000 FWHM (*m*/*z* = 200) at a range of 70–1050 *m*/*z* with an AGC Target setting at 3 × 10^6^. The fragment ion information of the top 6 precursors of each scan was acquired by data-dependent acquisition (DDA) at a mass resolution of 17,500 FWHM, and AGC Target of 1 × 10^5^.

For the identity of metabolites, the accurate *m*/*z* of precursors and product ions (MS/MS) were matched against an in-house standard library (containing information of retention time, accurate precursors and product ions), public databases including mzCloud, MoNA and LipidBlast, and finally the differential metabolites were manually checked by an experienced MS engineer.

### 2.5. Non-Targeted Metabolomics by GC-MS

A total of 20 μL of the 50% aqueous acetonitrile sample mentioned above and 10 μL 50 μg/mL of L-norleucine was combined and evaporated to dryness under nitrogen stream. The residue was reconstituted in 25 μL of 20 mg/mL methoxyamine hydrochloride in pyridine, and incubated at 37 °C for 90 min. A total of 25 μL of N,O-Bis(trimethylsilyl)trifluoroacetamide with 1% trimethylchlorosilane was added into the mixture and derivatized at 70 °C for 60 min prior to GC-MS metabolomics analysis.

Instrumental analysis was performed on the 7890A/5975C GC-MS system (Agilent Technologies Inc., Santa Clara, CA, USA) with a 5 MS Accent fused-silica capillary column (30 m × 0.25 mm × 0.25 μm; MACHEREY-NAGEL, Düren, GERMAN) to separate the derivatives. Carrier gas was helium (>99.999%) at a flow rate of 1 mL/min. The injection volume was 1 μL and the solvent delay time was 5.4 min. The initial oven temperature was held at 60 °C for 1 min, ramped to 240 °C at a rate of 12 °C/min, to 320 °C at 40 °C/min, and finally held at 320 °C for 4 min. The temperatures of injector, transfer line and electron impact ion source were set to 250 °C, 260 °C and 230 °C, respectively. The electron ionization (EI) energy was 70 eV, and the data were collected in full scan mode (*m*/*z* 50–600).

For the identification of differential metabolites performed by GC-MS, the AMDIS software was applied to deconvolute mass spectra from raw GC-MS data. Then, the purified mass spectra were automatically matched with an in-house standard library (containing information of retention time and mass spectra), Golm Metabolome Database and Agilent Fiehn GC/MS Metabolomics RTL Library.

### 2.6. RNA Sequencing Analysis

The retinas of five STZ-induced diabetic mice and three healthy mice were obtained, and the total RNA of retinas was extracted by using Trizol Reagent (Thermo Fisher). The transcriptome sequencing was performed with Genergy Biotechnology (Shanghai, China). The fastq reads were aligned to the mm10 mouse reference genome using STAR. The expression levels of genes were quantified by FeatureCounts. Differential expression analysis between groups was calculated using the DESeq2 R package. *p* < 0.05 and Fold Change > 1.2 or < 0.83 were the thresholds to identify differentially expressed genes (DEGs).

### 2.7. Genome-Wide Association Study (GWAS) Analysis

Single nucleotide polymorphism-trait associations (DM/DR-associated risk loci) were downloaded on 14 October 2022 from the GWAS Catalog with the keyword “diabetes mellitus” and “diabetic retinopathy”, respectively.

### 2.8. Statistics Analysis

When comparing the data of weight, blood glucose and OCT, the two-way ANOVA test and Tukey’s post hoc test were used with GraphPad Prism Software version 9.0. Other statistical analyses were performed using the R software (Version 4.2.0). According to the normality and homogeneity of variance, the *t* Test or Mann–Whitney U Test was used to obtain the differential metabolites between the two groups. Multivariate statistical methods, including principal component analysis (PCA) and orthogonal partial least squares discriminant analysis (OPLS-DA), were performed using the Ropls R package. A heatmap was conducted using MetaboAnalyst 4.0 (Xia Lab, McGill University, Montreal, QC, Canada) with the following parameter: data source, normalized data; clustering method, ward; carried out using the top 30 differential metabolites based on *t*-test/ANOVA. Enrichment analysis was based on the small molecule pathway database (SMPDB) by using MetaboAnalyst 4.0 (Xia Lab, McGill University, Montreal, Canada). Gene set enrichment analysis (GSEA) of the gene expression data based on the Kyoto Encyclopedia of Genes and Genomes (KEGG) was performed using ClusterProfiler R package (version 4.4.4) with the criteria of *p* < 0.05 and |normalized enrichment score| > 1.

## 3. Results

### 3.1. The Thickness of Inner Retina was Altered in STZ-Induced Mice

**Figure 1:**
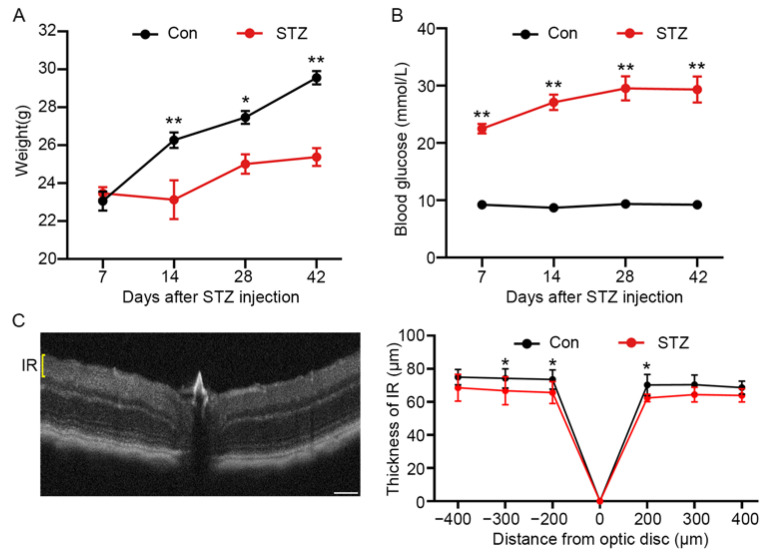
The establishment of STZ-induced diabetic mice retinas. (**A**) Body weight (g) of all mice from two groups. Data are presented as mean ± SEM, * *p* < 0.05, ** *p* < 0.01, *n* = 9 in the control group, *n* = 9 in the diabetic group. (**B**) Blood glucose (mmol/L) of all mice from two groups. Data are presented as mean ± SEM, * *p* < 0.05, ** *p* < 0.01, *n* = 9 in the control group, *n* = 9 in the diabetic group. (**C**) Representative image of a 15-week STZ-induced diabetic mouse retina captured using OCT showing the IR and quantification of 12 images from each group is represented in the line graph. The thickness of the inner retinal layer was reduced in the diabetic retina compared to the controls. Data are presented as mean ± SEM, *n* = 12 eyes per group. Scale bar, 120 μm. IR, inner retina.

To characterize the metabolic profile of the diabetic retina, we established a diabetic retinopathy mouse model induced by streptozotocin. Their blood glucose and fasting weight were measured weekly. The blood glucose level of diabetic mice was two- to three-fold greater than that of controls approximately 1 week after the last injection (9.20 ± 0.22 vs. 22.49 ± 0.83 at 1 week). Additionally, body weight decreased 12% compared with controls at 2 weeks after the onset of diabetes (26.27 ± 0.41 vs. 23.12 ± 1.02). (Figure 1). The reduction in thickness of diabetic retina layer can be measured via non-invasive OCT [22]. To evaluate the establishment of diabetic retinas, we performed OCT in STZ-induced diabetic mice and the controls to observe the structural change of retinas. As shown in Figure 1C, the thickness of the inner retina in diabetic mouse retina was reduced by 11% compared to controls 15 weeks post-injection (73.50 ± 1.68 vs. 65.33 ± 1.84). These observations suggest that retina damage has been caused by diabetes for 15 weeks in mice.

### 3.2. Metabolomics Displays Signatures of Metabolism Dysregulation in Diabetic Mice Retinas

**Figure 2:**
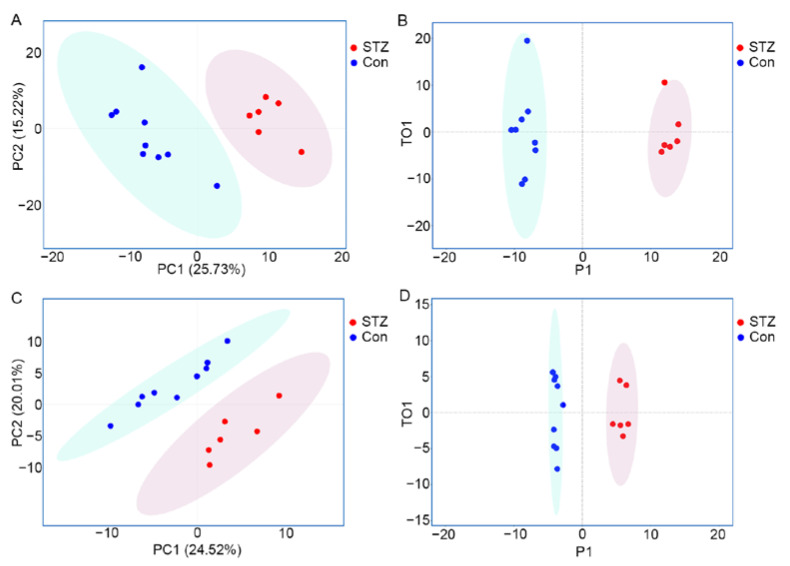
Principal component analysis of retinal metabolome profiles separates the STZ-induced DR group from controls. (**A**) Two-dimensional score plot of the PCA model comparing the control and STZ-induced diabetic group based on UHPLC-MS/MS; (**B**) Score plot of the OPLS-DA model based on UHPLC-MS/MS; (**C**) Two-dimensional score plot of the PCA model based on GC-MS; (**D**) Score plot of the OPLS-DA model based on GC-MS.

**Figure 3:**
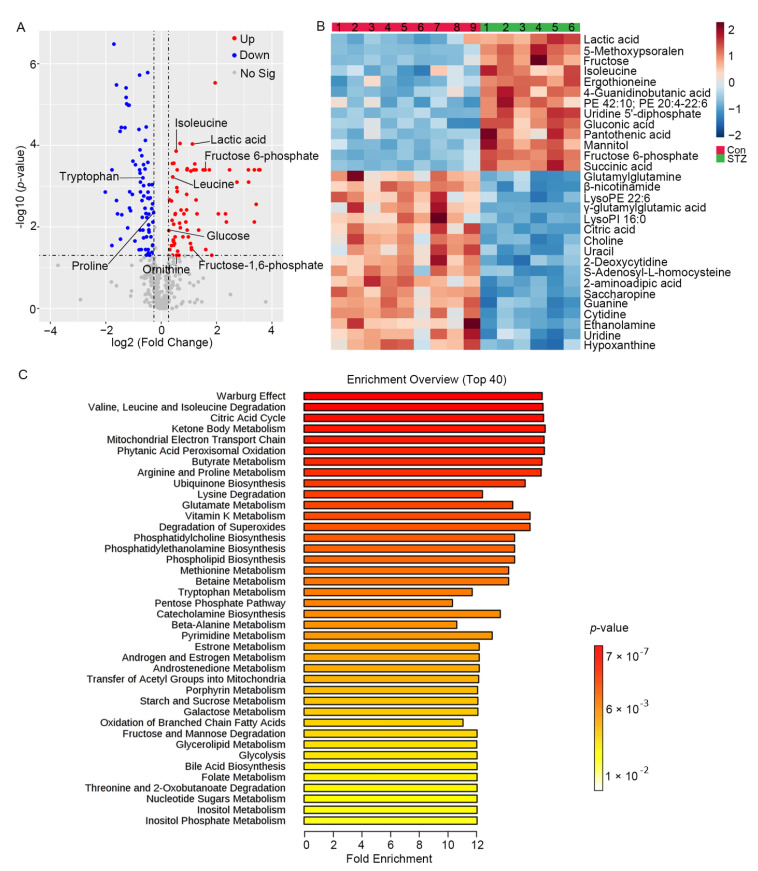
Metabolomics reveals differential metabolites and metabolic pathways in the diabetic retina of mice. (**A**) Volcano plot showing differential metabolites between two groups. Down-regulated and up-regulated metabolites are in blue and red, respectively. Non-significant metabolites are represented by gray dots (*p* < 0.05, fold change >1.2 or <0.83). (**B**) Heatmap showing relative peak areas of top 30 dysregulated metabolites in retina; (**C**) Metabolite set enrichment analysis showed top 40 differential pathways differed between the STZ-induced diabetic group and control group (*p* < 0.05).

After 16 weeks maintaining high blood glucose, the mice retinas of the STZ-induced diabetic group and control group were performed on the multiplatform-based metabolomics including UHPLC-MS/MS and GC-MS. In total, 417 metabolites were detected in the mice retina of the diabetic and control groups. PCA and OPLS-DA analysis showed a clear separation between the two groups (Figure 2). Using a *p* value of 0.05 for screening, 145 metabolites with fold change >1.20 or <0.83 were selected for further studies (Figure 3A). A total of 68 metabolites including glucose, fructose-6-phosphate, fructose-1,6-diphosphate, lactic acid, isoleucine, leucine and ornithine were significantly increased. Additionally, 77 metabolites, such as proline and tryptophan, were significantly decreased (Table S1). Figure 3B visualized the top 30 differential metabolites that distinguished the retina of diabetic mice from controls. To obtain the metabolic pathways associated with diabetic retinopathy, metabolite set enrichment analysis was further performed. A total of 50 metabolic pathways were identified, including the Warburg effect pathway, and valine, leucine and isoleucine degradation pathways—40 are presented in Figure 3C. These results indicate that metabolomics reveals several patterns of altered metabolites and related metabolism pathways that may help to identify pathogenic mechanisms in the diabetic retina of mice.

### 3.3. RNA-Sequencing Analysis Reveals the Alterations of Metabolic-Related Genes in the Diabetic Retinas of Mice

**Figure 4:**
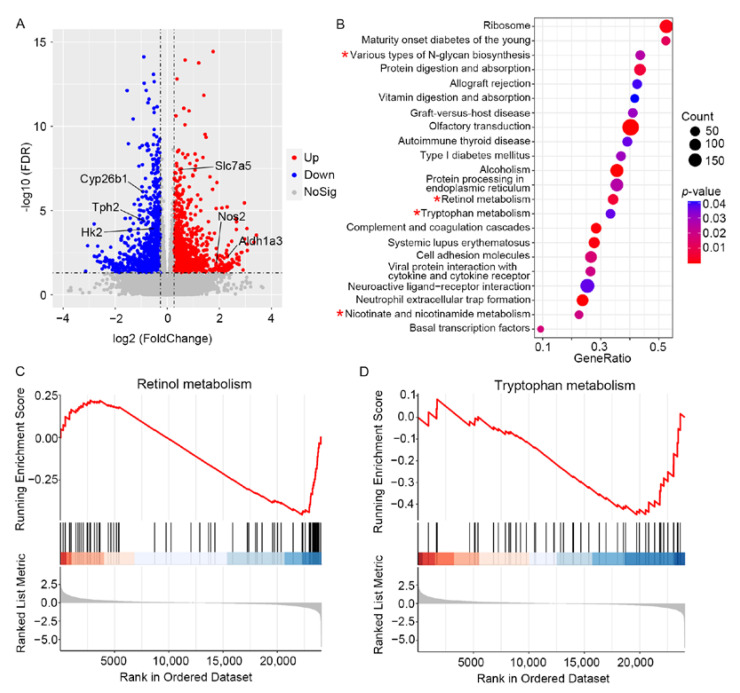
RNA-sequencing profiles demonstrated signatures of metabolism dysregulation in diabetic mice retina. (**A**) Volcano plot showing differentially expressed genes between two groups. Down-regulated and up-regulated metabolites are in blue and red, respectively. Non-significant metabolites are represented by gray dots (*p* < 0.05). (**B**) Gene set enrichment analysis showed that 22 differential pathways differed between the STZ-induced diabetic group and control group (*p* < 0.05, * metabolic pathway); (**C**,**D**) Two pathways were both enriched by metabolite and gene set enrichment analysis. (**C**) Gene set enrichment analysis of retinol metabolism. (**D**) Gene set enrichment analysis of tryptophan metabolism.

To further identify underlying regulators, RNA sequencing was performed to integrate metabolic enzyme alterations with metabolomics in STZ-induced diabetic retina. Analysis of the global transcriptional profiles of retina demonstrated that the retina of STZ-induced diabetic mice differed significantly in gene expressions. A total of 2521 differential expressed genes (DEG), with *p* value < 0.05 and fold change >1.20 or <0.83, were selected for further investigation. A total of 1315 DEGs including Aldh1a3, Slc7a5 and Nos2 were upregulated. Additionally, 1206 DEGs including Cyp26b1, Tph and Hk2 were down-regulated (Figure 4A, Table S1). Gene set enrichment analysis (GSEA) was performed to reveal master transcriptional regulators associated with metabolic pathways. A total of 22 pathways met the criteria of *p* < 0.05 and |normalized enrichment score| >1 (Figure 4B). Significant enrichments in four metabolic pathways including various types of N−glycan biosynthesis, retinol metabolism, tryptophan metabolism, and nicotinate and nicotinamide metabolism were collectively observed in STZ-induced diabetic retina (Figure 4B–D). Notably, retinol metabolism and tryptophan metabolism were also identified in differential metabolite set enrichment analysis. Overall, these results demonstrate that metabolic-related genes are altered at transcriptional levels in diabetic mouse retina.

### 3.4. Integrated Transcriptome–Metabolome Analysis Reveals the Characteristics of Metabolic Regulators in Diabetic Mouse Retina

To systematically evaluate the perturbed metabolism and potential regulators underlying STZ-induced diabetic retina, we jointly analyzed the featured metabolic pathways in metabolomics and the differentially expressed genes in transcriptomics. Through mapping the metabolic enzyme-related genes of 50 metabolic pathways enriched in the metabolomics of diabetic retinas, we found that 71 metabolic enzyme-related genes were differentially expressed at transcript levels in 16 out of 50 metabolic pathways (Table 1).

**Figure 5:**
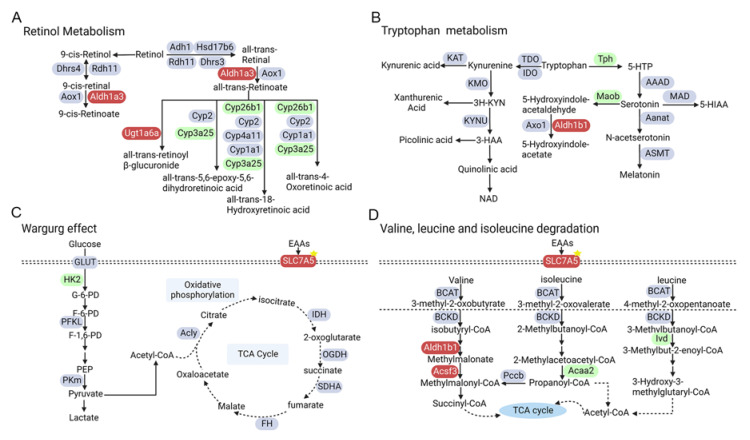
Overview of representative metabolic pathways with enzyme alterations at transcriptional levels in mice retina undergoing diabetic retinopathy. Down-regulated and up-regulated differential genes are in blue and red, respectively. (**A**) Retinol metabolism pathway; (**B**) Tryptophan metabolism pathway; (**C**) Warburg effect pathway; (**D**) Valine, leucine and isoleucine degradation pathway.

In addition, to further uncover the potential links between the identified differential metabolic enzyme genes and reported human genetics of diabetes or diabetic retinopathy, we quantified the overlap between the differential genes in the retina of STZ-induced diabetic mice and the candidate loci from genome-wide association study (GWAS), since genetic variants have been reproducibly associated with diabetic retinopathy through population-based GWAS [23,24]. Single nucleotide polymorphisms (SNPs) are the most commonly studied genetic variants in GWAS [25]. In our study, 6 out of 71 differentially expressed genes had records of SNPs associated with an increased risk of diabetes, including Uqcr10 and Slc7a5. As shown in Figure 5A,B, the retinol metabolism and tryptophan metabolism pathways, which are shared in transcriptome and metabolome, showed that the metabolites and differentially expressed metabolic enzyme-related genes increased (red color) and decreased (green color), respectively. In addition, the top two clustered metabolic pathways including the Warburg effect and valine, leucine and isoleucine degradation pathways from metabolomics analysis shared down-regulated metabolic genes (green) and up-regulated ones (red) with transcriptomes as well (Figure 5C,D). These results suggest that integrated metabolome–transcriptome analysis contributes to revealing alterations of metabolites and genes in several pathways including the Warburg effect, amino acid metabolism and retinol metabolism, which may provide potential metabolic regulators including Hk2 and Slc7a5 involved in DR.

## 4. Discussion

Currently, it is an important goal to identify potential intervention targets for diabetic retinopathy. There are unique metabolite profiles in human plasma and vitreous associated with diabetic retinopathy, which indicate underlying mechanisms and potential targets [6,26,27,28]. However, the characteristics of metabolites in retina have not been fully demonstrated. Additionally, there is a lack of integrated transcriptome–metabolome analysis of retina in diabetic mice. In this study, the metabolomics of diabetic mouse retina was performed using UHPLC-MS/MS and GC-MS technology. There were obvious differences in the metabolism profiles between the retina of controls and STZ-induced diabetic mice. After setting a *p* value of 0.05, a total of 145 metabolites were significantly altered in diabetic mice retinas with the fold change >1.2 or <0.83. Pathway enrichment analysis revealed that 50 metabolic pathways were altered in diabetic mice retina. Besides, RNA sequencing of diabetic mouse retina was also performed to explore the potential molecular mechanism of diabetic retinopathy. Integrated transcriptome–metabolome analysis revealed that 71 metabolic enzyme-related genes were expressed differentially in the retina of STZ-induced diabetic mice. Additionally, diabetes-associated SNPs within 6 out of 71 genes were identified based on the reported data of GWAS.

According to the metabolite set enrichment analysis, the top one pathway was the Warburg effect, which was first discovered in 1924 by Otto Warburg and has had an important effect on cancer metabolism [29]. Warburg proposed that, in the presence of cellular oxygen, cancer cells that synthesize adenosine triphosphate (ATP) undergo an energetic shift from oxidative phosphorylation to increased aerobic glycolysis. In our study, the metabolic pathway enrichment analysis implied that the Warburg effect may play pivotal roles in diabetic retinopathy (Figure 3C). Compared with controls, glycolytic metabolites including glucose, glucose 6-phosphate, fructose 6-phosphate, fructose 1, 6-diphosphate and lactic acid were up-regulated in diabetic mice retina (Figure 3A). Wang et al. reported that the combination of fructose 6-phosphate, lactic acid and two other metabolites displayed good discrimination between the control and DR patients [13]. Besides, Barba et al. demonstrated that glucose and lactic acid were increased in the vitreous of DR patients through 1H-NMR-based metabonomic approach [30]. Another study showed that lactic acid was also increased in the aqueous humor of DR patients via a 1H-NMR-based metabolomic approach [31]. These studies showed positive support for our animal model to explore the mechanism of DR. Hexokinase, converting glucose to glucose 6-phosphate, is an important enzyme in glycolysis. In our study, the mRNA expression of Hk2 was decreased in STZ-induced diabetic retina mice (Figure 4A), which was also decreased in the muscle of patients with T2DM [32]. Recently, several studies indicated that the glucose-induced stabilization of HK2 to proteolysis, driving HK2-linked glycolytic overload, could lead to increased oxidative stress and mitochondrial dysfunction [33,34]. Therefore, further validation for the protein expression, kinase activity, function and molecular mechanism of HK2 in diabetic retinopathy was necessary in the future. Nevertheless, the metabolomic and transcriptomic studies from mouse retina all indicate a key role for aerobic glycolysis to be critical in diabetic retinopathy.

We found that the levels of amino acids significantly differed in diabetic mouse retina compared with the controls (Figure 3A). For example, ornithine was significantly increased (citrulline was increased with *p* value = 0.053) and proline was decreased in diabetic mouse retina, which was similar with the plasma and vitreous metabolites of DR patients [13,27,35]. Moreover, the metabolic enzyme genes including nitric oxide synthase 2 (Nos2) were expressed differentially (Table 1). The results of metabolites and related genes reflect the upregulation of arginine metabolism. Liu et al. reported that microRNA-216a protects against human retinal microvascular endothelial cell injury in diabetic retinopathy via suppressing the NOS2/JAK/STAT axis [36]. The inhibitor of Nos2 has been reported to inhibit the development of diabetic retinopathy through reducing retinal microaneurysms, acellular capillaries and pericyte ghosts [37]. Additionally, the consistence of mouse retina and human samples indicate that STZ-induced diabetic mice could be used to further explore the molecular mechanism of arginine metabolism in regulating DR. Apart from arginine metabolism, the levels of leucine and isoleucine in branched-chain amino acid (BCAA) metabolism were also increased (valine was increased with *p* value = 0.06, Figure 3A). Moreover, high levels of leucine and isoleucine in the vitreous and plasma of DR populations were reported [7,13,38]. The increasing levels of circulating BCAAs may be related to the forceful neurotoxicity of glutamate in the retina, which plays a pivotal role in DR neurodegeneration [39]. Fundamentally, BCAAs were transported into cellular through SLC7A5 (Figure 5D), then could activate the mammalian target of the rapamycin (mTOR) pathway, which functions in the regulation of cell growth and proliferation [40]. Additionally, SNPs within SLC7A5 (rs76069656, rs4384608) were previously reported to link with an increased diabetes risk [23,24]. A recently published study reported that SLC7A5 could be inhibited by empagliflozin, which ameliorates diabetic retinopathy manifestations [41]. Therefore, the levels of amino acids should be paid more attention to, which would contribute to understanding the pathogenesis and exploring the potential therapeutic targets of DR.

The tryptophan metabolism pathway was enriched simultaneously in metabolomics and transcriptomics. Additionally, tryptophan is metabolized primarily to kynurenine by indoleamine 2,2-dioxygenase (IDO). In our study, the level of tryptophan was significantly decreased in diabetic mouse retina compared to the controls (Figure 3A). It was reported that the level of plasma kynurenine was increased, and level of plasma tryptophan was decreased in patients with DR [42]. Additionally, the altered tryptophan–kynurenine metabolism pathway may play a role in the pathogenesis of diabetic cataracts [42]. Recently, the importance of the tryptophan–kynurenine metabolism pathway has been demonstrated in animal models of diabetes, but not in diabetic retinopathy [43], whereas the difference in the kynurenine level was not statistically significant in our study. Moreover, the expression of IDO1 and IDO2 displayed no significant difference between the two groups (Table 1). According to our results, we inferred that the circulating tryptophan metabolites nor the local tryptophan metabolites may participate in the pathogenesis of DR. Thus, further exploration of tryptophan metabolism is necessary for the mechanism and treatment of DR.

Another pathway enriched in both metabolomics and transcriptomics is the all-trans-retinol or vitamin A metabolism pathway, which has been reported to play an important role in regulating obesity and insulin resistance [44]. Aldh1a3 (Raldh3) is one of only three retinaldehyde dehydrogenases that catalyze the final oxidative step from retinaldehyde to retinoic acid. Then, retinoic acid was catalyzed by cytochrome P450, family 26 (CYP26s) to more polar compounds which could be eliminated in urine and feces after glucuronidation. The differences in enzymes in retinol metabolism were explored in our results. The expression of Aldh1a3 was increased in the diabetic group, whereas the level of CYP26s was decreased, which could cause the accumulation of all-trans-retinoic acid. Retinoic acid could regulate cell differentiation, apoptosis and cell cycle arrest through activating retinoic acid receptors and retinoid X receptors [45]. However, retinoic acid was not identified in our metabolites. Additionally, apart from regulating the aforementioned biological synthesis of retinoic acid, Aldh1a3 also participates in other metabolic pathways, including glycolysis and amino acid metabolism. Although a higher dietary intake of retinol is associated with a lower risk of diabetic retinopathy [46], the roles of metabolites and enzymes in retinol metabolism pathway have not been fully demonstrated in DR. Validation and exploration are still required in the future.

There are some directions that could be further researched in the future. First, our focus metabolic pathways are limited, which are composed of the top two pathways enriched in the small molecule pathway database (SMPDB) and two pathways that cross with GSEA. We readily acknowledge that many data remain unexplored, and this dataset provides rich information of potential pathogenesis for DR. For example, genes such as Aldh1a3, Cyp26b1 and Acaa2, without reference regarding anti-diabetic retinopathy function, would be the candidates for our future exploration. In spite of this, further validation is still necessary and required.

## 5. Conclusions

We used an integrated multi-omics approach and identified disproportionate differences between STZ-induced diabetic mouse retina and the controls. These data revealed the disturbance of the Warburg effect, amino acids and retinol metabolism, which may provide a basis for exploring the underlying metabolic mechanisms and therapeutic potentials of diabetes retinopathy.

## Figures and Tables

**Table 1:** Differentially expressed metabolic enzyme-related genes via integrating transcriptome–metabolome analysis.

Metabolic Pathway	Differentially Expressed Gene
Warburg Effect	Hk2, Hras, Kit, Met, Nras, Ntrk1, Pgam1, Slc7a5 *, Trp53
Valine, leucine and isoleucine degradation	Acaa2, Acsf3, Aldh1b1, Ivd
Oxidative phosphorylation	Atp6v0c, Atp6v1f, Cox6b1, Cycs, Ndufb2, Ndufb3, Ndufv3, Ppa1, Tcirg1, Uqcr10 *, Uqcrb
Arginine and proline metabolism	Aldh4a1, Azin2, Ckmt1, Lap3, Maob, Nos2, Smox
Alanine, aspartate and glutamate metabolism	Asns
Cysteine and methionine metabolism	Bhmt, Cbs, Mat1a, Mri1, Mtap^*^
Tryptophan metabolism	Tph2
Pentose phosphate pathway	Rgn
Pyrimidine metabolism	Cda, Cmpk2, Ctps, Ctps2, Dhodh, Entpd3
Starch and sucrose metabolism	Pygm
Glycerolipid metabolism	Agpat1 *, Agpat4, Dgkb^*^, Lipg, Lpin3, Mboat1 *, Plpp2, Pnliprp2
Glycolysis	Aldh3a1, Pck1, Pgk1
Inositol Phosphate Metabolism	Ipmk, Pip4k2a
Sphingolipid metabolism	9130409I23Rik, Asah2, Hexa, Neu3, Smpd1
Steroid biosynthesis	Lss, Nsdhl
Retinol metabolism	Aldh1a3, Aldh1a7, Cyp26b1, Cyp3a25, Ugt1a6a

Genes labeled with * had records of single nucleotide polymorphisms (SNPs) associated with an increased risk of diabetes.

## Data Availability

The data presented in this study are available in the article.

## Supplementary Materials

The following supporting information can be downloaded at: https://www.mdpi.com/article/10.3390/metabo12121245/s1, Table S1: Summary table of key abnormal biochemical changes and morphological changes.
