# Supplementary material for: Integrated Metabolomics and Transcriptomics Reveal Metabolic Patterns in Retina of STZ-Induced Diabetic Retinopathy Mouse Model
Source: Metabolites. 2022 Dec 9;12(12):1245. doi: 10.3390/metabo12121245 (PMC9782096; doi:10.3390/metabo12121245)
Supplement: Supplementary file 1 [file metabolites-12-01245-s001.zip › metabolites-2052584-supplementary.pdf]

Table S1. Summary table of key abnormal biochemical changes and morphological changes

Category	Name	Fold change (STZ vs Con)	*P* value
Metabolites	Glucose	1.212	0.012
	Glucose 6-phosphate	5.000	0.005
	Fructose 6-phosphate	3.001	0.0004
	Fructose 1, 6-diphosphate	2.132	0.036
	Ornithine	1.470	0.049
	Proline	0.830	0.004
	Arginine	0.947	0.526
	Leucine	1.335	0.0006
	Isoleucine	1.453	0.0001
	Valine	1.354	0.072
	Tryptophan	0.633	0.0006
	Kynurenine	0.913	0.955
Enzyme genes	Hk2	0.750	0.00009
	Nos2	3.660	0.017
	Slc7a5	1.390	3.805E-08
	Aldh1a3	4.900	0.006
	Tph2	0.530	0.00004
	Cyp26b1	0.520	7.444E-07
OCT	Inner retina thickness	0.889	<0.05

STZ, streptozotocin; Con, control; OCT, optical coherence tomography
